# Community Pharmacy Service for Patients With Inhaled Medications: A Multi‐Perspective Observation and Assessment Under Routine Conditions

**DOI:** 10.1111/jep.70271

**Published:** 2025-09-08

**Authors:** Ann‐Christin Kroenert, Lucas Freyberg, Claudia Sehmisch, Sebastian Michael, Thilo Bertsche

**Affiliations:** ^1^ Department of Clinical Pharmacy, Institute of Pharmacy, Medical Faculty Leipzig University Leipzig Germany; ^2^ Drug Safety Center Leipzig University Leipzig Germany; ^3^ Saxon Pharmacists' Association Saxony Germany; ^4^ Albert‐Schweitzer‐Pharmacy Leipzig Germany; ^5^ Löwen‐Pharmacy Waldheim Germany

**Keywords:** community pharmacy, community pharmacy services, dry powder inhalers, inhalation, metered dose inhalers, pharmacist, respiratory tract diseases

## Abstract

**Rationale:**

Outcome studies have shown the benefits of inhalation consultations. Therefore, the service of inhalation consultations from pharmacists has been implemented in guidelines. Recently, this service became reimbursable for German community pharmacies.

**Aims:**

We aimed to investigate how this service is performed under routine conditions to actually achieve the proven benefits.

**Methods:**

We evaluated the reimbursed inhalation service under routine conditions in community pharmacies in Saxony, Germany. An external trained monitor observed routine services and documented predefined handling errors in patients' inhalation demonstrations. Besides, the monitor checked which contents of predefined checklists were addressed. After the consultations, patients and pharmaceutical staff were asked about the pervious service via a questionnaire.

**Results:**

We analysed 48 reimbursed inhalation services in 13 different community pharmacies. Most consultations were on metered dose inhalers and dry powder inhalers, with 42% (20/48) each. We observed a median of *n* = 2 handling errors per patient (Q25: 1; Q75: 3). On average, 77% of those two errors were addressed by the pharmaceutical staff during the following consultation (95% confidence interval [69%; 86%]; minimum: 0%; maximum: 100%). Overall, patients' contentment with the service was very high (overall median: 5), while pharmaceutical staff's contentment was high (overall median: 4). There was no correlation between duration and unaddressed errors (*r* = 0.16; *p* = 0.35) or patients' contentment (*r* = 0.19; *p* = 0.26).

**Conclusion:**

Handling errors in patients' inhalation technique were common but mostly recognized and addressed by the pharmaceutical staff during the reimbursed inhalation service. Patients' contentment with the service was high to very high, and they were usually more content with the service than the pharmaceutical staff.

**Abbrevations**
DPI: dry powder inhaler
MDI: metered dose inhaler
MDI‐breath: breath induced metered dose inhaler
SMI: soft mist inhaler

## 1 Introduction

Chronic respiratory diseases belong to the most frequent chronic noncommunicable diseases affecting over 540 million people worldwide and causing over 3 million deaths each year. This makes them the third leading cause of death [1]. In Germany, the prevalence of chronic obstructive pulmonary disease is 8.5% in people aged 45 years and older and for asthma, 8% in people aged 18 years and older [2]. The prevalence of chronic respiratory diseases has increased rapidly over the last years [1]. Dramatically, many deaths due to chronic respiratory diseases could have been prevented by good adherence to the available treatment options [3]. Effective medications are available and can be escalated according to the disease stages [4]. Therefore, pulmonologists recently defined symptom prevention and disease remission as top priority therapy goals [5].

Medications for chronic respiratory diseases are frequently available in an inhaled administration form that combines good local efficacy with few systemic risks. However, this goes along with complex inhalation devices which have a particular high potential for handling errors [6]. Additionally, the inhalation technique differs fundamentally between devices [7]. Understanding and performing the right inhalation technique is highly important to get a sufficient amount of active ingredient to the local site of action and to prevent systemic adverse drug reactions [8].

Therefore, complex inhaled dosage forms require comprehensive pharmaceutical consultation. Studies have already proven the benefits of pharmaceutical care under structured conditions in improving asthma‐specific quality of life, knowledge, medication adherence, asthma severity, peak expiratory flow and patients' inhalation technique [9, 10, 11, 12, 13]. The pharmaceutical care needs to be offered repeatedly as most patients tend to develop handling errors within 6 months after consultation [14]. Moreover, patients trust community pharmacists and wish for the involvement of community pharmacists into the regular chronic respiratory disease care [15]. Consequently, the role of the pharmacist in the care of asthma patients should be expanded alongside that of the physician. Therefore, recently pharmacists have been even explicitly mentioned in asthma guidelines [16]. Moreover, a community‐pharmacy‐service concerning inhalation consultation has been implemented as a reimbursed service in Germany in June 2022 [17]. This service can be provided to patients aged 6 years and older who do not take part in disease management programmes, got a new device or had their last inhalation consultation for their current device at least 1 year ago. The service is not limited to certain diseases. It can be provided by all pharmaceutical staff working in community pharmacies. The service can be repeated every 12 months [17]. It is remunerated via the night‐and‐emergency fund. The service consists of an inhalation demonstration by the patient followed by an inhalation consultation. Additionally, preparation and subsequent follow‐up and invoicing is performed. Therefore, the word ‘service’ is used to describe the reimbursed inhalation service as a whole. When specifically talking about the direct patient consultation as part of this service, the word ‘consultation’ is used. The consultation does not include the time required for preparation, subsequent follow‐up and invoicing.

So far, little attention has been paid to the question of how this service is performed under routine conditions. Significant improvements in patient‐reported outcomes (e.g., asthma‐specific quality of life, self‐efficacy, knowledge and medication adherence) were reported [9, 11]. Additionally, patients' asthma severity, self‐reported symptoms, peak expiratory flow and inhaler technique have been improved [9, 11]. However, a systematic comparison in the literature addressing external and internal assessments of the reimbursed inhalation service is lacking.

Therefore, we aimed to assess the reimbursed inhalation service under routine conditions. We monitored the on‐site service in community pharmacies using predefined checklists. Additionally, we asked the patients and the pharmaceutical staff who conducted the service for their opinions on the service.

## 2 Methods

### 2.1 Ethics Approval

This study has been approved by the ethics committee of the Medical Faculty of the University of Leipzig on 24 October 2023 (#344/23‐ek). Participating community pharmacies and patients were informed about data safety and pseudonymization before the start of the study. Patients had to give their written informed consent in advance.

### 2.2 Participants and Setting

In November 2023, all community pharmacies in Saxony were invited to participate in this survey via a newsletter from the Saxon Pharmacists' Association. Additionally, as a reminder, community pharmacies were contacted via email, telephone and in person. When the community pharmacies agreed to participate, they received flyers to promote the study. They were asked to recruit up to 10 patients for an ‘Action Day’ for reimbursed inhalation services between 7 November 2023 and 30 April 2024. On this day, a monitor of the study team visited the community pharmacy. The pharmaceutical staff of the corresponding community pharmacy performed a community pharmacy service with their patient under routine conditions. The monitor observed this service by using validated checklists. Afterwards, patients and pharmaceutical staff members were separately questioned about their evaluation of the previous service.

If the pharmaceutical staff had special training for inhalation consultation those training was voluntarily and not in the context of this study. As to observe the consultations under routine conditions, the study team did not provide any training or written instructions.

We aimed to observe services under routine conditions during a predefined period of time to estimate the prevalence. Therefore, we included every community pharmacy and every patient who wanted to participate during our study period and had not calculated a special sample size in advance. A post hoc sample size calculation using calculator.net [18] revealed a margin of error of 10%. We used the following parameters: Sample size: 46; population proportion: 87% of the patients having handling errors; confidence level: 95%; population size: over 6,000,000 people [2].

### 2.3 Inclusion Criteria for Community Pharmacies

All invited community pharmacies had to be located in Saxony and had to offer the reimbursed inhalation service.

It did not matter if the participating pharmacies offered the service before the study or had their first reimbursed service during the study.

### 2.4 Inclusion and Exclusion Criteria for Patients

The patients who volunteered to participate in the study had to give their written informed consent in advance. They had to be at least 18 years old. Their need for inhalation therapy must have been diagnosed at least 1 year ago, and they should be using an inhaler at the moment. Patients with all diseases treated with inhaled medication could participate.

Additionally, they had to match the inclusion criteria for the reimbursed service.

Patients who were not able to give their written informed consent, who could not understand the study cognitively or the language, as well as those who were under 18 years old, pregnant or breastfeeding, were excluded.

### 2.5 Study Design

The community pharmacies recruited their patients for an ‘Action Day’ for the reimbursed inhalation service which was observed by a monitor, who is a pharmacist, a member of the study team and independent from the corresponding community pharmacy. Therefore, the study was carried out in a four‐step process:

1. The first step was to identify the number of handling errors per patient. After patients gave their written informed consent, they were invited to perform an inhalation demonstration. To identify and document inappropriate inhalation techniques, this demonstration was observed by the monitor using a modified form of the ‘checklist—correct use of inhalation medication’ by Hämmerlein et al. [19] with 17 predefined handling errors (S1). This includes both the technical steps on the device and the handling by the patients themselves before, during and after the inhalation. Noncongruent single procedures were labelled as handling errors.
2. The second step was to evaluate which of the patient's handling errors were addressed during the following inhalation consultation. Therefore, the pharmaceutical staff of the corresponding community pharmacy performed a routine inhalation consultation. This consultation was observed by the external monitor to identify and document the process. Therefore, the monitor from the study team filled out a second checklist, the checklist provided by the German Federal Chamber of Pharmacists, that is mandatory when carrying out the service [20] (S2).
3. The third step was to compare the external observation with the internal assessment of patient and pharmaceutical staff to check if the content was understandable. For this, pharmaceutical staff and patient were separated after the consultation into different rooms. Both got a comparable checklist to the one that was used to observe patient's inhalation technique to recall what has been handled during the consultation (S3).
4. Additionally, the contentment of patients and pharmaceutical staff with the service was assessed by using matching questionnaires designed by the study team (S4, S5).

Furthermore, the duration of the consultation was measured by the external monitor to investigate the correlation between duration, unaddressed handling errors and contentment of the patients by using Spearman's correlation.

To be able to match the checklists and questionnaires later on, the pairs of patients and the corresponding pharmaceutical staff members were given a pseudonym.

### 2.6 Statistics and Data Evaluation

The answers were entered into Microsoft Office Excel 2019 MSO (16.0.10409.20028) 64‐bit and IBM SPSS Statistics Version 29.0 for data analysis. As descriptive statistical methods, we used median with 25%‐ and 75%‐quantiles, for durations mean with 95% confidence interval (CI) and percentages for relative numbers. For describing correlations, Spearman's correlation with a 95% CI was used.

A kappa test (using Cohen's kappa coefficient) was performed to evaluate the interrater reliability. Therefore, some randomly selected services have been observed by two monitors from the study team simultaneously. Both filled in the checklist for patient's inhalation technique (S1) and the checklist for the inhalation consultation (S2) simultaneously but independently from each other. After this, the checklists have been compared by using a kappa test.

The STROBE Statement for cross‐sectional studies was used as a reporting guideline when writing this paper [21].

## 3 Results

### 3.1 Observation Quality

The mean Cohen's kappa coefficient for the checklist for the inhalation consultation was 0.84 (95% CI [0.71; 0.96]) over all questions of the checklist. For the checklist for the inhalation technique of the patient, the mean Cohen's kappa was 0.71 (95% CI [0.59; 0.83]).

### 3.2 Participants

**Figure 1:**
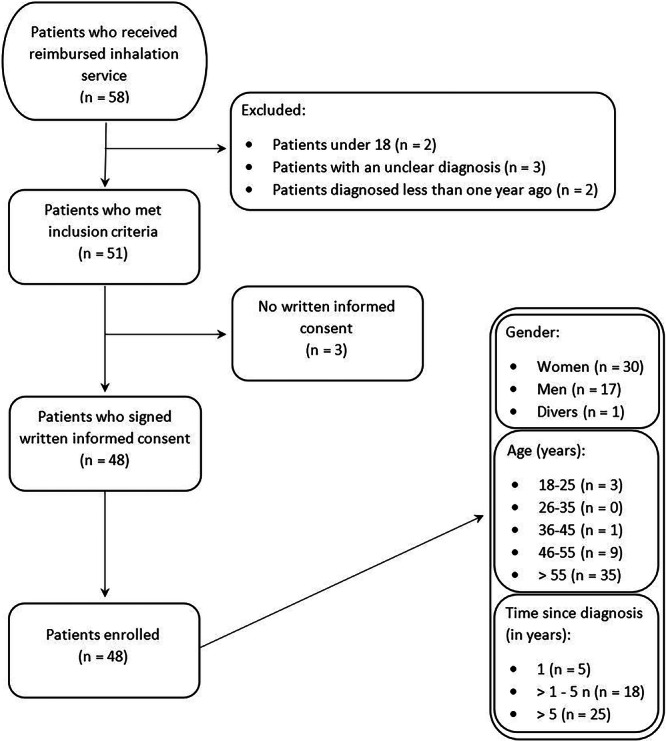
Enrolment and data of participating patients.

In total, 58 patients received a reimbursed inhalation service in 13 different community pharmacies. Forty‐eight of those patients fulfilled all inclusion criteria and gave their written informed consent to participate (Figure 1). The inhalation demonstration from patient's inhalation technique was observed in 46 processes, because two patients got new devices so they were not able to demonstrate their inhalation technique before the inhalation consultation.

The consultations have been provided by a total of 29 different pharmaceutical staff members, of 40% (19/48) were pharmacists, 44% (21/48) were pharmaceutical technical assistants, 6% (3/48) were pharmaceutical engineers and 10% (5/48) were persons of other professions. Fifty‐eight percent (28/48) of the pharmaceutical staff had voluntary advanced training for inhalation consultations before providing the service.

Forty‐two percent (20/48) of services concerned metered dose inhalers (MDI) and another 42% (20/48) dry powder inhalers (DPI). Eight percent (4/48) of the services have been about soft mist inhalers (SMI), 6% (3/48) concerning MDI with spacer and 2% (1/48) about a breath‐induced MDI (MDI‐breath).

### 3.3 Identified Handling Errors in Patient's Inhalation Technique and Which Were Addressed During Inhalation Consultation

Before the consultations, in median 2 handling errors (Q25: 1, Q75: 3; minimum: 0, maximum: 6) occurred per patient. Thirteen percent (6/46) of the patients' inhalation procedures were without any handling errors.

**Figure 2:**
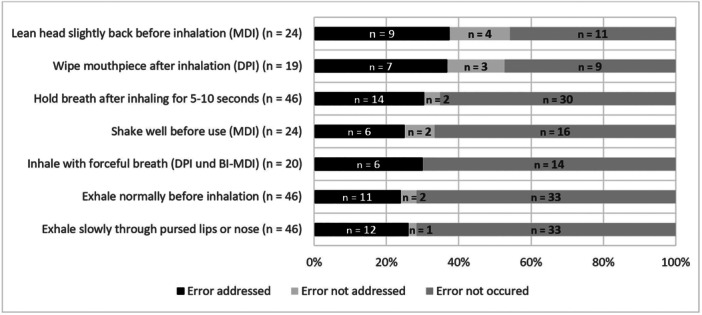
Most frequently identified predefined handling errors in patient's inhalation technique and how often they were addressed during the following inhalation consultation. For metered dose inhalers (MDI) and dry powder inhalers (DPI), specific points of the two most common handling errors are shown. For general points on inhalation technique, the three most common handling errors are shown as two had the same values.

Figure 2 shows the most common handling errors identified before the inhalation consultation. Additionally, the figure shows whether the handling errors were subsequently addressed during the consultation. From the median of *n* = 2 handling errors per patient, on average, 77% were addressed during the following consultation (95% CI [69%; 86%]; minimum: 0%, maximum: 100%). In 21 consultations, 100% of patients' handling errors were addressed.

### 3.4 Recall of Inhalation Consultation From Three Perspectives

**Figure 3:**
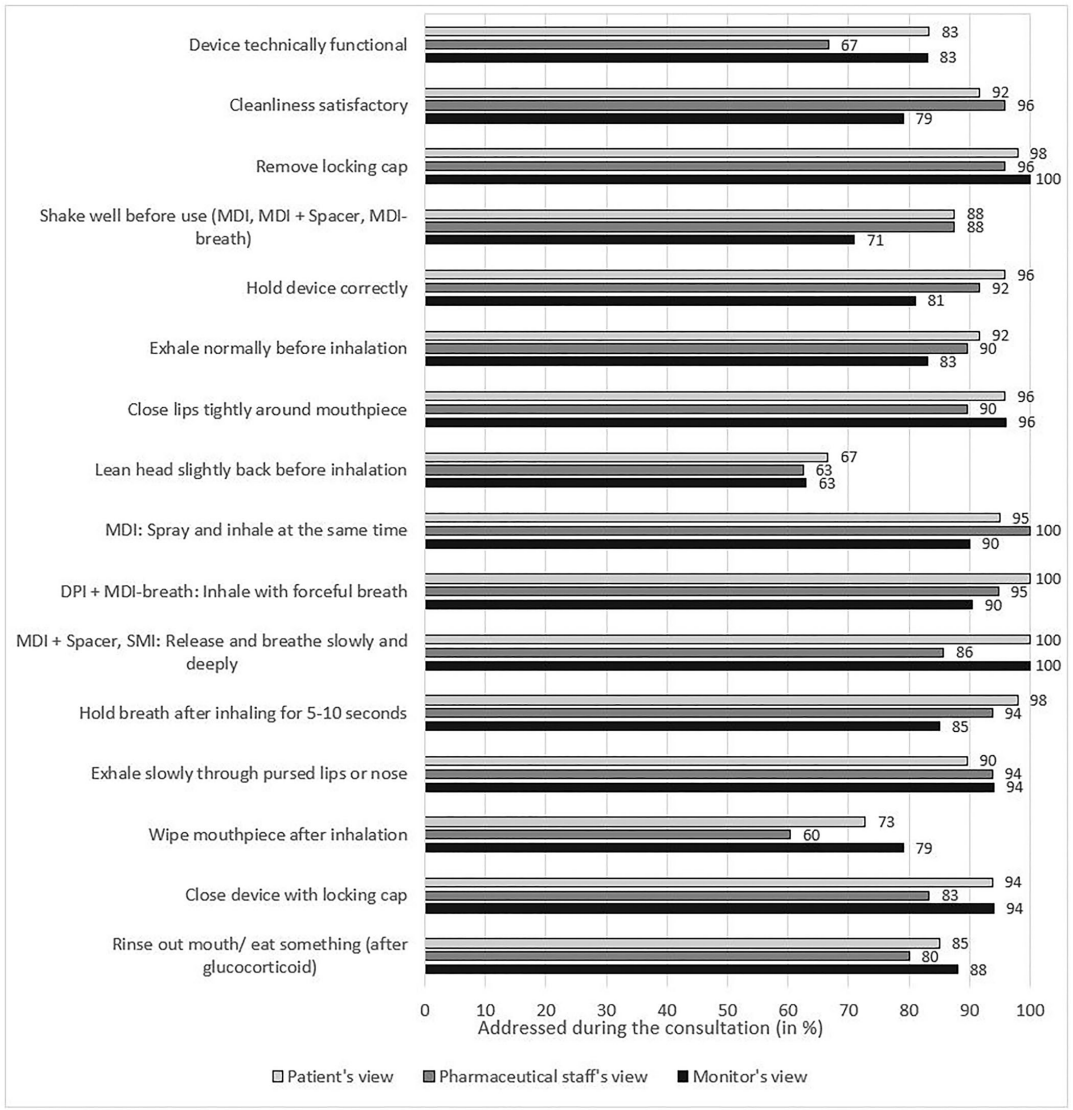
Patient's, pharmaceutical staff's and monitor's assessment on what was addressed during the inhalation consultation.

Figure 3 shows the points that patients and pharmaceutical staff remembered after the inhalation consultations. Additionally, the assessment by the external monitor is shown.

### 3.5 Contentment of Patients and Pharmaceutical Staff

**Figure 4:**
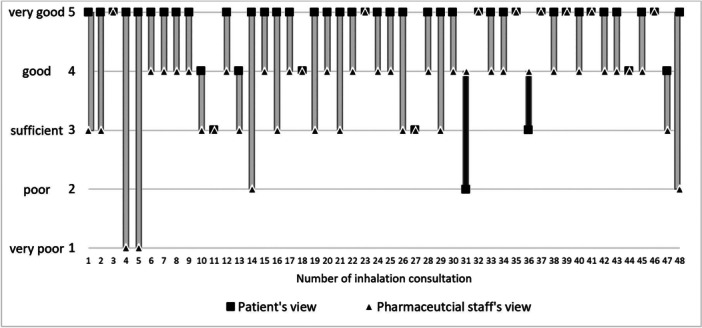
Patient's and pharmaceutical staff's assessment of the question ‘Was the inhalation service helpful to improve the understanding and the technique of the correct use of the inhaler?’ Results for the other questions can be seen in S6.

Figure 4 shows that 71% of the patients (34/48) rated the improvement of their understanding of the inhalation technique higher than the pharmaceutical staff did. In 25% (12/48) of patients and pharmaceutical staff rated equally, and in 4% (2/48) of patients rated the improvement of their understanding worse than the pharmaceutical staff.

Overall, patients' contentment with the service was very high (overall median: 5), while pharmaceutical staff's contentment was high (overall median: 4). The results and median values of all questions are shown in S6.

### 3.6 Duration of the Inhalation Consultation

**Figure 5:**
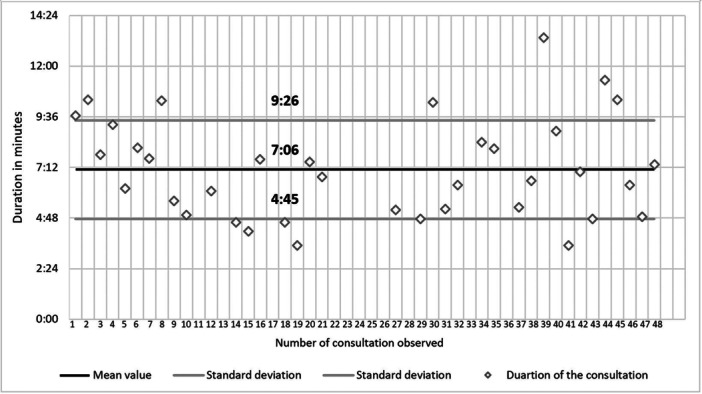
Durations of direct patient consultations as part of the reimbursed inhalation service.

Figure 5 shows the duration of the inhalation consultations. The consultations lasted between 3:30 and 13:20 min. On average, the time required for the direct patient consultation was 7:06 min (95% CI [6:21 min; 7:51 min]).

We found no correlation between the duration of consultation and the number of unaddressed handling errors in total (*r* = 0.039; *p* = 0.823, 95% CI [−0.303; 0.371]) or the number of unaddressed handling errors in relation to the number of handling errors identified during patient's inhalation demonstration (*r* = 0.006; *p* = 0.973, 95% CI [−0.332; 0.343]). Moreover, no correlation was found between the duration of the consultation and the number of mentioned points during the consultation (*r* = 0.140; *p* = 0.416, 95% CI [−0.207; 0.456]).

Additionally, no correlation was found between the median overall contentment of the patients and the duration of the consultation (*r* = 0.073; *p* = 0.670, 95% CI [−0.271; 0.401]).

## 4 Discussion

### 4.1 Main Aspects

In our study, we investigated routine reimbursed inhalation services in community pharmacies in Saxony, Germany. We observed numerous handling errors in patients' inhalation technique. Although, a large proportion of these handling errors were consecutively addressed during inhalation consultations by the pharmaceutical staff, some handling errors remained unaddressed in the current consultation. However, patients remembered counselling content that, according to the monitor, was not addressed in the current consultation. In general, patients' overall contentment with the service was high to very high. The patients themselves generally rated the service quality higher than the pharmaceutical staff. What is more, a good consultation does not necessarily have to take a long time since no correlation was found between the duration and the unaddressed handling errors or patients' contentment. All those findings provide useful information for optimizing the reimbursed inhalation service in the future.

### 4.2 Methodological Aspects

There were slightly more women than men, and many more people older than 55 than younger people. However, this group of patients is representative for the related disease patterns since no differences in asthma depending on age are known and more women are suffering from asthma [22]. Additionally, the prevalence for chronic obstructive pulmonary disease increases in older patients [2].

The kappa test for the observation of the inhalation consultation revealed an almost perfect agreement between the two monitors and, therefore, a very good interrater reliability.

The consultations were held by pharmaceutical staff of different qualifications on a wide range of different inhalation devices indicating a good generalizability of our study.

### 4.3 Identified Handling Errors in Patients' Inhalation Technique

The most frequently identified handling errors show which aspects of the inhalation technique should be focused on during a patient‐oriented and prioritized service. In our study, in 13% of the demonstrations, no predefined handling errors at all were identified. Some other studies reported this number to be lower when, for example, 7.2% of patients treated with MDI expressed handling errors [23]. Others described the number as higher when 23.1% of inhalation processes were performed without handling errors [24].

The common handling errors found in this study were rated by an expert panel to have a medium to high clinical risk factor [25]. For example, not shaking the MDI containing a suspension before use could lead to over‐ or underdosing in all following inhalations [25] and to a up to 50% reduced lung deposition [26]. Additionally, inhaling slowly instead of forcefully with a DPI can lead to reduced lung drug delivery and therefore to reduced efficacy [26]. Exhaling before inhalation and holding breath after inhalation are rated by Neininger et al. to have a medium to high clinical risk [25], but Melani et al. found no impact of those errors on the lung drug delivery [26].

This shows that at least some common handling errors found in this study have high clinical relevance and need to be solved.

### 4.4 Addressed Handling Errors During Inhalation Consultations

To meet the special needs of every single patient, we intended to know whether the handling errors that actually occurred in the inhalation demonstration were recognized and solved during the consultation. We consider this to be very relevant, as there is hardly any time under routine conditions to always carry out a comprehensive service programme in its entirety without necessity. It is, in contrast, more resource‐efficient if the reimbursed inhalation service is tailored to individual patients' needs.

The special technique for DPI to inhale with a forceful inhalation was always addressed when a handling error occurred. All points of the inhalation technique in general were addressed in over 80% of the consultations, where the handling errors occurred. In former studies, patients' inhalation technique has been improved by about 90% through pharmaceutical counselling [10].

Prioritization and not to overwhelm the patient with too many topics at once, may provide a good explanation why some of the identified handling errors in our external observation were not the subject of the consultations.

### 4.5 Recall of the Inhalation Consultation From Three Perspectives

Our study was characterized by the fact that we also interviewed the patients and the pharmaceutical staff directly after the external observation. This allowed us to determine the extent to which the patients remembered the content.

Patients remembered some items not addressed during the current consultation (S8, Table 1). This phenomenon might suggest a recall bias. Eventually, patients thought those points were addressed during the current consultation, but they gained their knowledge from former consultations. The majority of the points recalled by the patients that were not addressed during the current consultation depend to the most common handling errors found in this study. This indicates that patients have the knowledge of how to use the inhaler but have problems in practically performing the inhalation technique correctly. This shows the importance of practical training and of demonstrating the inhalation technique to the patients instead of only explaining it.

Additionally, patients did not remember the point ‘exhaling slowly through pursed lips or nose’. Pharmaceutical staff, on the other hand, remembered this point in every procedure, they addressed it according to the monitor. This indicates that the didactics of patient teaching can be improved (S8, Table 3).

The pharmaceutical staff, on the other hand, often thought that they did not address some points properly, which were actually addressed (S8, Table 2). A study from Bosnia and Herzegovina was the other way round, where pharmacists overrated themselves [27]. A review concluded that the self‐assessment depends on the actual competency. While pharmacists with high competency underrated their knowledge, pharmacists with lower competency overrated their own knowledge [28]. This indicates the high competency of the pharmacists in this study.

### 4.6 Contentment of Patients and Pharmaceutical Staff

In general, patients wish for the involvement of pharmacists in their chronic respiratory disease management, especially concerning, for example, the provision of information about the disease and appropriate medication and review and correction of their inhalation technique. Patients also expressed a desire for pharmacists to be competent, friendly, empathic and attentive [29].

In our study, we focused on the patients' direct evaluation of the current service. Fulfilling patients' needs seems to us to be a key parameter, especially when used in routine care.

On average patients rated their contentment with good to very good (S6). This goes along with the satisfaction of patients with inhalation consultations and knowledge of the pharmacists in a long‐term pharmaceutical management in Australia [30, 31].

Additionally, we asked pharmaceutical staff for their contentment with the service in a corresponding questionnaire. Overall, the pharmaceutical staff rated the service as good. Those results show that patients are more content with the advice they receive and that pharmaceutical staff underestimate their work.

### 4.7 Duration of the Inhalation Consultation

The average time in our study—only for inhalation consultation as part of the whole service—was about 7 min. Reimbursed inhalation service is estimated to take around 25 min and costs 20 euros [17, 32]. The duration of direct patient consultations appears comparatively short but clearly underestimates the actual effort required for the service, as time for consultation has to be supplemented by time, for example, for preparation, documentation and invoicing. Taking up almost half of the time that is reimbursed for the service in total, the consultation should not take much longer. Additionally, the calculation of the remuneration was based on the assumption that 80% of the services were provided by pharmaceutical technical assistants and only 20% by pharmacists [32]. However, our study showed that both groups provide the same number of consultations. Since many pharmacies apparently see the need to involve a pharmacist in certain consultation situations, this calculation should be reconsidered.

We found no correlation between time and unaddressed handling errors in the inhalation consultation or the overall contentment of the patients. This shows the sophisticated knowledge of the pharmaceutical staff in patients' needs. The lack of correlation between duration and the number of mentioned points during the consultation shows that consultations are highly individual and mentioning one point can take a different length of time according to the individual patient's needs. Additionally, we believe that it is not always necessary to mention all points listed on the checklist to perform good counselling. The number of unaddressed handling errors in relation to the number of handling errors identified during patient's inhalation demonstration seems sensible, as specifically mentioning the existing handling errors instead of all points seems necessary to increase patient's safety.

### 4.8 Recommendations for Future Optimization of Reimbursed Inhalation Service

Regarding the most common handling errors found in this study and the points most often forgotten by the patients after the consultation, memory cards for patients showing the most important steps of the inhalation process might be helpful. Especially for the MDI, the pharmaceutical staff could attach a ‘shake before use’ button to patient's inhaler.

Additionally, it seems to be useful to highlight the most common and the most unaddressed handling errors on pharmaceutical staff's checklist for the reimbursed inhalation service and to address them more explicitly in their advanced training. Moreover, memory cards containing the most overseen handling errors might be helpful for the pharmaceutical staff to recall them during consultations.

Otherwise, to make services more effective, it may be helpful to point out more clearly that the checklist [20] is intended to be used for the observation of patients' inhalation technique. Thereby pharmacists can recognize handling errors more easily and mention only the most important points instead of mentioning all points listed on the checklist.

Previous studies already showed this so called ‘teach‐back’‐method is effective but needs to be more specified [33].

Moreover, former studies indicated that handling errors return after an individual amount of time but maximum after 6 months [14]. Further studies should investigate sustained effects under real‐life conditions with an eye on whether the once‐yearly service is sufficient or if it should be adaptable to individual patient's needs.

### 4.9 Limitations

In our study, the following limitations should be considered while interpreting and extrapolating the results: When assessing the lack of solutions to some handling errors in the consultations, it should be noted that we did not evaluate which points had already been addressed in previous consultations. We can, within this study, only include the observable handling errors in the respective consultations, but not the knowledge that the local pharmaceutical staff may have gained from previous contacts with their patients. Not addressing some handling errors in consultations should not be considered as inadequate advice, as addressing only the most clinically relevant handling errors may be better in order not to overwhelm the patient. These important decisions should be made individually by the pharmaceutical staff.

The patients consistently rated the advice as positive, even if a certain bias is to be assumed due to the inclusion of patients with a positive attitude. Additionally, it can be assumed that the pharmacies that voluntarily participated in this study also spent a considerable amount of time on preparation, follow‐up and detailed training not even asked for in this study.

The duration assessed in our study only included the direct patient consultation. Preparation (including also educational sessions), follow‐up and bureaucracy have not been considered in this context. Therefore, our data regarding the duration of inhalation consultations should not be used, for example, for economic evaluations.

Finally, this study only examined the short‐term benefits for patients in terms of resolving the handling errors identified. Long‐term effects on disease‐ or quality‐of‐life‐related parameters would require a long‐term follow‐up.

## 5 Conclusion

We found that handling errors were frequent even in long term users of inhalation devices. Many of those handling errors were directly addressed by the pharmaceutical staff during the consultation. In addition, the patients remembered a substantial number of items—even those that were not actually addressed during the current consultation. Additionally, patients were very content with the service.

Moreover, even a limited duration for the consultation seems to be effective to solve identified handling errors. Nevertheless, it should not be underestimated that the actual time required for the service is significantly longer than the actual time required for the direct patient consultation.

The results indicate that patients derive at least a short‐term benefit from the service but repetitive follow‐up appointments may be useful to solve handling errors comprehensively and sustainably.

## Author Contributions

A.C.K., L.F. and T.B. made contributions to the conception and design of the work and this article, and the analysis and interpretation of data for this article. L.F., C.S. and S.M. recruited community pharmacies for participation. C.S. and S.M. recruited patients for participation in their own community pharmacies. L.F. and A.C.K. observed the routine services as external monitors. All authors finally approved the version to be published. All authors agreed to be accountable for all aspects of the work in ensuring that questions related to the accuracy or integrity of any part of the work are appropriately investigated and resolved.

## Ethics Statement

This study has been approved by the ethics committee of the Medical Faculty of the University of Leipzig in October 2023 (#344/23‐ek). Participating community pharmacies and patients were informed about data safety and pseudonymous data collection before the start of the study. Patients had to give their written informed consent.

## Conflicts of Interest

The authors declare no conflicts of interest.

## Supporting information

Supplement 1 Checklist to observe patient's inhalation technique.

Supplement 2 Checklist for inhalation consultation.

Supplement 3 Recall of inhalation consultation.

Supplement 4 Questionnaire patient.

Supplement 5 Questionnaire pharmaceutical staff.

Supplement 6 Contentment with the service from patients and pharmaceutical staff.

Supplement 7 ‐ Handling errors grouped by inhaler.

Supplement 8 ‐ Recall of addressed points.

## Data Availability

The data sets generated and analysed during the current study are available from the corresponding author on reasonable request.
