# Supplementary material for: Community Pharmacy Service for Patients With Inhaled Medications: A Multi‐Perspective Observation and Assessment Under Routine Conditions
Source: J Eval Clin Pract. 2025 Sep 8;31(6):e70271. doi: 10.1111/jep.70271 (PMC12416124; doi:10.1111/jep.70271)
Supplement: Supplementary file 1 — Supplement 1 Checklist to observe patient's inhalation technique. [file JEP-31-0-s001.pdf]

Checklist to observe patient's inhalation technique

Device	Metered Dose Inhaler (MDI) ☐	Dry Powder Inhaler (DPI) ☐
	Breath-induced Metered Dose Inhaler (MDI-breath) ☐	Other ☐

Demonstration of the inhalation

Condition of the device		Correct	Incorrect
**1.** Device technically functional and components fit together		☐	☐
**2.** Cleanliness satisfactory		☐	☐
**Preparation of the inhalation**			
**3.** Remove locking cap		☐	☐
**4.** Shake well before use (Usually for MDI, MDI + Spacer, MDI-breath)		☐	☐
**5.** Further steps to make device ready to use (e.g., pull lever, attach spacer)		☐	☐
**Inhalation process**			
**6.** Hold device correctly (MDI, MDI-breath: vertically; DPI: usually horizontally)		☐	☐
**7.** Exhale normally		☐	☐
**8.** Close lips tightly around mouthpiece		☐	☐
**9.** Lean head slightly back (MDI)		☐	☐
**10.**	MDI: Spray and inhale at the same time, as exception also for Jethaler® (DPI)	☐	☐
	MDI-breath, DPI: Inhale with forceful breath	☐	☐
	MDI + Spacer: Release and breathe slowly and deeply	☐	☐
**11.** Inhale slowly and deeply (MDI) or quickly and deeply (DPI)		☐	☐
**12.** Hold breath after inhaling (5-10 sec.)		☐	☐
**13.** Exhale slowly through pursed lips or nose		☐	☐
**14.** Avoid exhaling into device		☐	☐
**15.** Wipe mouthpiece (DPI)		☐	☐
**16.** Close device with locking cap		☐	☐
**17.** Rinse out mouth/ eat something (after glucocorticoid)		☐	☐

Modified from "checklist – correct use of inhalation medication" Hämmerlein A, Müller U, Schulz M. Pharmacist-led intervention study to improve inhalation technique in asthma and COPD patients. J Eval Clin Pract 2011; 17(1): 61–70 DOI: 10.1111/j.1365-2753.2010.01369.x
