# Supplementary material for: Community Pharmacy Service for Patients With Inhaled Medications: A Multi‐Perspective Observation and Assessment Under Routine Conditions
Source: J Eval Clin Pract. 2025 Sep 8;31(6):e70271. doi: 10.1111/jep.70271 (PMC12416124; doi:10.1111/jep.70271)
Supplement: Supplementary file 2 — Supplement 2 Checklist for inhalation consultation. [file JEP-31-0-s004.pdf]

■ Work material for community pharmacy services

Community pharmacy service concerning inhalation consultation

**Pharmacy:**		**Date:**	
**Pseudonym:**			

**Device**	
☐ Metered Dose Inhaler (MDI)	☐ Dry Powder Inhaler (DPI)
☐ Breath-induced MDI (MDI-breath)	☐ Soft Mist Inhaler (SMI) (Respimat®)
☐ MDI with Spacer (MDI+S)	Other:

	**Content of the inhalation consultation**	**Was trained**
	**Condition of the device**	
1	Device technically functional and components fit together	☐
2	Cleanliness satisfactory	☐
	**Preparation**	
3	Shake well before use (Usually for MDI, MDI + Spacer, MDI-breath)	☐
4	Remove locking cap	☐
5	Further steps to make device ready to use (e.g., insert cartridge, pull lever, attach spacer, release active ingredient)	☐
	**Inhalation**	
6	Hold device correctly (MDI, MDI+Spacer: Mouthpiece down; MDI-breath: Vertically; DPI: Vertically or horizontally, not shaking; SMI: Horizontally)	☐
7	Exhale completely (not in device)	☐
8	Close lips tightly around mouthpiece	☐
9	Hold head upright	☐
10	MDI: Spray and simultaneously breathe deeply, slowly and long	☐
	MDI-breath: Breathe deeply, slowly and long (until maximum inspiration) do not cover air hole	☐
	MDI+S: Release in spacer and immediately breath slowly and deeply (< 3-5 sec.)	☐
	DPI: Inhale deeply and fastly (until maximum inspiration)	☐
	SMI: Release and simultaneously breathe deeply, slowly and long, do not cover air hole	☐
11	Hold breath for 5-10 seconds	☐
12	Exhale slowly through pursed lips or nose (avoid exhaling into device)	☐
13	Control success (taste, sound, counter, empty capsule)	☐
	**Finish**	
14	Wipe mouthpiece	☐
15	Reset device (MDI-breath: Ease, eventually remove empty capsule)	☐
16	Attach locking cap	☐
17	Rinse out mouth or eat something after using a glucocorticoid	☐
	**Other** (and duration)	

BAK

German Version: Copyright Bundesapothekerkammer

Stand: 16.06.2023

English translation of:
Bundesapothekerkammer (BAK) Arbeitsmaterialien für die pharmazeutischen Dienstleistungen, Erweiterte Einweisung in die korrekte Arzneimittelanwendung mit Üben der Inhalationstechnik - Checkliste zur korrekten Anwendung inhalativer Arzneimittel, Stand 16.06.2023 [Internet]. [cited 2025, Feb 10]. Available from: https://www.abda.de/fileadmin/user_upload/assets/Pharmazeutische_Dienstleistungen/pDL/Inhalativa/pDL_Inhalativa_Arbeitshilfe_CL_BAK.pdf.
