# Supplementary material for: Community Pharmacy Service for Patients With Inhaled Medications: A Multi‐Perspective Observation and Assessment Under Routine Conditions
Source: J Eval Clin Pract. 2025 Sep 8;31(6):e70271. doi: 10.1111/jep.70271 (PMC12416124; doi:10.1111/jep.70271)
Supplement: Supplementary file 3 — Supplement 3 Recall of inhalation consultation. [file JEP-31-0-s008.pdf]

Recall of the inhalation consultation

Demonstration of the inhalation

Condition of the device	Was very well trained	Was sufficient trained	Was not trained
**1.** Device technically functional and components fit together	☐	☐	☐
**2.** Cleanliness satisfactory	☐	☐	☐
**Preparation of the inhalation**			
**3.** Remove locking cap	☐	☐	☐
**4.** Shake well before use (Usually for MDI, MDI + Spacer, MDI-breath)	☐	☐	☐
**5.** Further steps to make device ready to use (e.g., pull lever, attach spacer)	☐	☐	☐
**Inhalation process**			
**6.** Hold device correctly (MDI, MDI-breath: vertically; DPI: usually horizontally)	☐	☐	☐
**7.** Exhale normally	☐	☐	☐
**8.** Close lips tightly around mouthpiece	☐	☐	☐
**9.** Lean head slightly back (MDI)	☐	☐	☐
**10.** MDI: Spray and inhale at the same time, as exception also for Jethaler® (DPI)	☐	☐	☐
MDI-breath, DPI: Inhale with forceful breaths	☐	☐	☐
MDI + Spacer: Release and breath slowly and deeply	☐	☐	☐
**11.** Inhale slowly and deeply (MDI) or quickly and deeply (DPI)	☐	☐	☐
**12.** Hold breath after inhaling (5-10 sec.)	☐	☐	☐
**13.** Exhale through pursed lips or nose	☐	☐	☐
**14.** Avoid exhaling into device	☐	☐	☐
**15.** Wipe mouthpiece (DPI)	☐	☐	☐
**16.** Close device with locking cap	☐	☐	☐
**17.** Rinse out mouth/ eat something (after glucocorticoid)	☐	☐	☐

Modified from ”checklist – correct use of inhalation medication” Hämmerlein A, Müller U, Schulz M. Pharmacist-led intervention study to improve inhalation technique in asthma and COPD patients. J Eval Clin Pract 2011; 17(1): 61–70 DOI: 10.1111/j.1365-2753.2010.01369.x
