# Supplementary material for: Community Pharmacy Service for Patients With Inhaled Medications: A Multi‐Perspective Observation and Assessment Under Routine Conditions
Source: J Eval Clin Pract. 2025 Sep 8;31(6):e70271. doi: 10.1111/jep.70271 (PMC12416124; doi:10.1111/jep.70271)
Supplement: Supplementary file 4 — Supplement 4 Questionnaire patient. [file JEP-31-0-s002.pdf]

Contentment with the community pharmacy service concerning inhalation consultation

Questionnaire Patient

Thank you for taking time to complete this questionnaire, that helps to improve the community pharmacy services and make them more sustainable. Your answers will be treated trustworthy and will only be used for scientific evaluations. The results will be evaluated pseudonymously.

1. Which gender do you identify with?

☐ Men ☐ Women ☐ Diverse

2. How old are you?

☐ Under 18-25 ☐ 26-35 ☐ 36-45 ☐ 46-55 ☐ Over 55 years

3. When have you been diagnosed?

☐ Less than one year ago ☐ 1 – 5 years ago ☐ More than 5 years ago

4. Please rate the competency of the pharmaceutical staff in providing the inhalation service.

Very poor ☐ 1 ☐ 2 ☐ 3 ☐ 4 ☐ 5 Very good

5. Has the pharmaceutical staff considered your individual needs and questions during the inhalation service?

Very poor ☐ 1 ☐ 2 ☐ 3 ☐ 4 ☐ 5 Very good

6. How good has the pharmaceutical staff provided the required knowledge concerning the usage of the inhaled medication?

Very poor ☐ 1 ☐ 2 ☐ 3 ☐ 4 ☐ 5 Very good

7. Was the inhalation service helpful to improve your understanding and technique of the correct use of the inhaler?

Very poor ☐ 1 ☐ 2 ☐ 3 ☐ 4 ☐ 5 Very good

8. How do you rate the short-term benefit of this inhalation service regarding your health and understanding of the medication?

Very poor ☐ 1 ☐ 2 ☐ 3 ☐ 4 ☐ 5 Very good

8.1 Which of the following aspects have been improved by the inhalation service? (Multiple answers possible)

☐ Inhalation technique
☐ Knowing the most important steps of the inhalation process
☐ Improved knowledge about the disease
☐ Taking drugs more often
☐ Knowledge about the structure of the device

9. Desired improvements: Please let us know which improvements concerning this service you would like to see and which further services concerning your medication would be useful.

____

Thank you for your participation!
