# Supplementary material for: Community Pharmacy Service for Patients With Inhaled Medications: A Multi‐Perspective Observation and Assessment Under Routine Conditions
Source: J Eval Clin Pract. 2025 Sep 8;31(6):e70271. doi: 10.1111/jep.70271 (PMC12416124; doi:10.1111/jep.70271)
Supplement: Supplementary file 5 — Supplement 5 Questionnaire pharmaceutical staff. [file JEP-31-0-s003.pdf]

Contentment with the community pharmacy service concerning inhalation consultation

Questionnaire Pharmaceutical staff

Thank you for taking time to complete this questionnaire, that helps to improve the community pharmacy services and make them more sustainable. Your answers will be treated trustworthy and will only be used for scientific evaluations. The results will be evaluated pseudonymously.

1. What is your profession?

☐ Pharmacist ☐ Pharmaceutical technical assistant ☐ Pharmaceutical engineer ☐ Other

2. Have you completed advanced training concerning inhaled medication before providing this service?

☐ Yes ☐ No

3. Please rate your own competency in providing the inhalation service.

Very poor ☐ 1 ☐ 2 ☐ 3 ☐ 4 ☐ 5 Very good

4. Have you considered the individual patient's needs and questions during the inhalation service?

Very poor ☐ 1 ☐ 2 ☐ 3 ☐ 4 ☐ 5 Very good

5. How good have you provided the required knowledge concerning the usage of the inhaled medication?

Very poor ☐ 1 ☐ 2 ☐ 3 ☐ 4 ☐ 5 Very good

6. Was the inhalation service helpful to improve patient's understanding and technique of the correct use of the inhaler?

Very poor ☐ 1 ☐ 2 ☐ 3 ☐ 4 ☐ 5 Very good

7. How do you rate the short-term benefit of this inhalation service for the patient?

Very poor ☐ 1 ☐ 2 ☐ 3 ☐ 4 ☐ 5 Very good

7.1 Which of the following aspects have been improved for the patient by the inhalation service? (Multiple answers possible)

☐ Inhalation technique
☐ Knowing the most important steps of the inhalation process
☐ Improved knowledge about the disease
☐ Adherence
☐ Knowledge about the structure of the device

8. Desired improvements: Please let us know which improvements concerning this service you would like to see and which further resources you think are necessary when providing the service to meet patient's needs and to improve the quality of the service.

____

Thank you for your participation!
