# Supplementary material for: Community Pharmacy Service for Patients With Inhaled Medications: A Multi‐Perspective Observation and Assessment Under Routine Conditions
Source: J Eval Clin Pract. 2025 Sep 8;31(6):e70271. doi: 10.1111/jep.70271 (PMC12416124; doi:10.1111/jep.70271)
Supplement: Supplementary file 6 — Supplement 6 Contentment with the service from patients and pharmaceutical staff. [file JEP-31-0-s005.pdf]

Table: Median contentment with the service from patients and pharmaceutical staff on a range from 1 (very poor) to 5 (very good) with 25%- and 75%-quantile in brackets

Question	Patient's assessment (median)	Pharmaceutical staff's assessment (median)
**Please rate the competency of the pharmaceutical staff in providing the inhalation service**	5 (Q_{25}= 5; Q_{75}= 5)	4 (Q_{25}= 4; Q_{75}= 4)
**Has the pharmaceutical staff met the individual patient's needs during the inhalation service?**	5 (Q_{25}= 5; Q_{75}= 5)	4 (Q_{25}= 4; Q_{75}= 5)
**How good has the pharmaceutical staff provided the required knowledge concerning the usage of the inhaled medication?**	5 (Q_{25}= 5; Q_{75}= 5)	4 (Q_{25}= 4; Q_{75}= 4)
**Was the inhalation service helpful to improve the understanding and the technique of the correct use of the inhaler?**	5 (Q_{25}= 5; Q_{75}= 5)	4 (Q_{25}= 3; Q_{75}= 4)
**How do you rate the short-term benefit of this inhalation service regarding health and understanding of the medication?**	5 (Q_{25}= 4; Q_{75}= 5)	5 (Q_{25}= 4; Q_{75}= 5)
**Overall contentment (median of all five questions asked)**	5 (Q_{25}= 5; Q_{75}= 5)	4 (Q_{25}= 4; Q_{75}= 5)
