# Supplementary material for: Community Pharmacy Service for Patients With Inhaled Medications: A Multi‐Perspective Observation and Assessment Under Routine Conditions
Source: J Eval Clin Pract. 2025 Sep 8;31(6):e70271. doi: 10.1111/jep.70271 (PMC12416124; doi:10.1111/jep.70271)
Supplement: Supplementary file 7 — Supplement 7 ‐ Handling errors grouped by inhaler. [file JEP-31-0-s006.pdf]

Table: Handling errors grouped by inhaler

	Metered Dose Inhaler (MDI) (n = 24)			*Dry Powder Inhaler (DPI) (n = 19)*			*Soft Mist Inhaler (SMI) (n = 3)*		
Handling Error	Addressed	Not addressed	Not occurred	Addressed	Not addressed	Not occurred	Addressed	Not addressed	Not occurred
Lean head slightly back before inhalation	9 (37 %)	4 (17 %)	11 (46 %)	NA	NA	NA	NA	NA	NA
Wipe mouthpiece after inhalation	NA	NA	NA	7 (37 %)	3 (16 %)	9 (47 %)	NA	NA	NA
Hold breath after inhaling for 5-10 seconds	6 (25 %)	0	18 (75 %)	7 (37 %)	1 (5 %)	11 (58 %)	1 (33 %)	1 (33 %)	1 (33 %)
Shake well before use	6 (25 %)	2 (8 %)	16 (67 %)	NA	NA	NA	NA	NA	NA
Inhale with forceful breath	NA	NA	NA	6 (30 %) *	0	14 (70 %) *	NA	NA	NA
Exhale normally before inhalation	5 (21 %)	1 (4 %)	18 (75 %)	6 (32 %)	1 (5 %)	12 (63 %)	0	0	3 (100 %)
Exhale slowly through pursed lips or nose	7 (29 %)	0	17 (71 %)	4 (21 %)	1 (5 %)	14 (74 %)	1 (33 %)	0	2 (66 %)

NA = not applicable
* For this handling error n = 20 as it is relevant for breath induced MDI (BI-MDI) as well.
