# Supplementary material for: Community Pharmacy Service for Patients With Inhaled Medications: A Multi‐Perspective Observation and Assessment Under Routine Conditions
Source: J Eval Clin Pract. 2025 Sep 8;31(6):e70271. doi: 10.1111/jep.70271 (PMC12416124; doi:10.1111/jep.70271)
Supplement: Supplementary file 8 — Supplement 8 ‐ Recall of addressed points. [file JEP-31-0-s007.pdf]

Table 1: Points patients thought were addressed during the current consultation but that were not addresses according to the monitor and the pharmaceutical staff (in %)

Points potentially addressed	Patient's assessment	Pharmaceutical staff's assessment	Monitor's assessment
Hold device correctly	96	92	81
Exhale normally before inhalation	92	90	83
Lean head slightly back before inhalation	67	63	63
Inhale with forceful breath (DPI, MDI-breath)	100	95	90
Hold breath after inhaling for 5 – 10 seconds	98	94	85

Table 2: Points that were addressed during the current consultation according to patients and the monitor but pharmaceutical staff could not remember (in %)

Points potentially addressed	Patient's assessment	Pharmaceutical staff's assessment	Monitor's assessment
Device technically functional	83	67	83
Close lips tightly around mouthpiece	96	90	96
Release and breath slowly and deeply (SMI, MDI + Spacer)	100	86	100
Close device with locking cap	94	83	94

Table 3: Points that were addresses during the current consultation according to the monitor and the pharmaceutical staff but patients could not remember (in %)

Points potentially addressed	Patient's assessment	Pharmaceutical staff's assessment	Monitor's assessment
Exhale slowly through pursed lips or nose	90	94	94
